# EffiShapeFormer: Shapelet-Based Sensor Time Series Classification with Dual Filtering and Convolutional-Inverted Attention

**DOI:** 10.3390/s26010307

**Published:** 2026-01-03

**Authors:** Junjie Bao, Shengcai Wang, Xuehai Tang, Shuaiqin Zhang, Hui Wang, Lei Wang, Qianxi Zhang, Nengchao Wu, Xinyu Yang, Xianyu Zhang, Xiaofeng Li, Jun Liao, Li Liu

**Affiliations:** 1 School of Mechanical Engineering, Xinjiang University, Urumqi 830017, China; 107552404289@stu.xju.edu.cn (J.B.); 107552404177@stu.xju.edu.cn (S.W.); zhangqianxi@stu.xju.edu.cn (Q.Z.); wnc@stu.xju.edu.cn (N.W.);; 2 United Laboratories of TT&C and Communication, Korla 841001, China; 3 School of Big Data & Software Engineering, Chongqing University, Chongqing 400030, China

**Keywords:** sensors data, time series classification, shapelet, Shapeformer, attention

## Abstract

In the field of sensors, time series classification holds significant importance for applications such as industrial monitoring, mechanical fault diagnosis, and action recognition. However, while existing models demonstrate excellent classification accuracy, they generally suffer from insufficient interpretability. Shapelet-based methods offer interpretability advantages, yet existing models like ShapeFormer suffer from high computational resource consumption and low training efficiency during shapelet discovery and training phases, limiting their applicability in complex sensor time series classification tasks. To address this, our research proposes Efficiency ShapeFormer (EffiShapeFormer), an efficient time series classification framework, based on the latest shapelet model ShapeFormer. During the Shapelet Discovery phase, EffiShapeFormer introduces a dual-filtering mechanism. The Coarse Screening module efficiently identifies discriminative shapelets, while the Class-specific Representation module models these features to extract class-specific characteristics. Subsequently, in the Generic Representation stage, the proposed Convolution-Inverted Attention (CIA) module achieves synergistic integration of local feature extraction and global dependency modeling to capture cross-category generic features. Finally, the model fuses class-specific and generic features to achieve efficient and accurate time series classification. Experimental results on 22 sensor time series datasets demonstrate that EffiShapeFormer achieves higher average accuracy and F1-scores than baseline models, validating the proposed method’s significant advantages in both efficiency and performance.

## 1. Introduction

Time series data, as a fundamental and ubiquitous data form, underpins a wide range of domains and is inherently characterized by its sequential order and temporal dependency. Among various data mining tasks, Time Series Classification (TSC) has gradually become a prominent research focus. In particular, time series data generated from sensors are widely utilized in industrial monitoring [1], mechanical fault diagnosis [2], environmental sensing [3], and action recognition [4]. However, sensor signals often exhibit complex characteristics such as pronounced long-range dependencies, non-stationarity, multi-scale structures, and heavy noise contamination. These challenges arise from the intricate intrinsic physical mechanisms underlying signal generation, substantial external environmental disturbances, and the coupling effects among multiple sensors [5]. Such characteristics not only hinder traditional time series processing methods from effectively capturing discriminative representations, but also pose severe challenges to the representation capacity and generalization performance of current deep learning models for sensor-based time series. Moreover, under resource-constrained computational scenarios, the demands for both model efficiency and interpretability become increasingly critical. Consequently, numerous approaches have been proposed in recent years to address the TSC problem more effectively. In 2017, Vaswani et al. introduced the groundbreaking Transformer [6] architecture—an entirely self-attention-based sequence modeling framework.Its success is largely attributed to high parallelism and strong global feature modeling capability. The Transformer’s strong ability to model global dependencies has also garnered widespread attention in the field of time series analysis.

However, despite the Transformer architecture’s excellence in capturing long-range dependencies and global semantic information, its direct application to time series tasks still encounters several challenges. The computational complexity of the standard self-attention mechanism grows quadratically with the sequence length, making it inefficient for modeling long time series. Moreover, inherent properties such as trend, periodicity, and multi-scale structures limit the effectiveness of purely global attention mechanisms. To overcome these limitations, numerous Transformer-based variants have been proposed to enhance temporal modeling efficiency and performance, including Informer [7], Reformer [8], Autoformer [9], Crossformer [10], and LightTS [11]. These models introduce mechanisms such as sparse attention, sequence decomposition, and architectural optimization, achieving remarkable improvements in long-sequence prediction and modeling tasks. Nevertheless, despite their advances in temporal dependency modeling, these methods remain limited in time series classification. Although they exhibit strong global modeling capabilities, their attention weights fail to directly indicate the discriminative importance of input features, resulting in limited interpretability. Consequently, it becomes difficult to uncover the decision-making rationale of such models in sensor signal analysis. To address these issues, researchers have begun exploring alternative frameworks that balance interpretability and classification performance for complex sensor-based time series data.

To balance model performance and interpretability, researchers have recently explored hybrid architectures that integrate traditional temporal feature extraction techniques with deep representation learning [12]. Compared to deep attention-based models, the Shapelet approach has attracted considerable attention due to its strong interpretability and outstanding discriminative capability [13,14]. Shapelets are short, highly class-discriminative subsequences that capture locally discriminative morphological variations and have been shown to play a pivotal role in time series classification [15,16].

Nevertheless, traditional Shapelet discovery methods typically depend on exhaustive searches and repeated distance computations across a vast number of candidate subsequences, leading to extremely high computational complexity and significant resource consumption [17]. This greatly restricts their applicability to high-dimensional and large-scale sensor datasets. To improve efficiency, ShapeFormer enhances the Offline Shapelet Discovery (OSD) [18] process, reducing the time required for shapelet extraction to a certain extent, while achieving remarkable temporal modeling performance through the integration of a Transformer architecture. However, when applied to sensor time series data characterized by strong noise, multi-scale structures, and non-stationary dynamics, ShapeFormer still encounters substantial computational overhead and distance metric costs during candidate evaluation. Furthermore, its standard Transformer encoder retains quadratic complexity with respect to sequence length, resulting in heavy training burdens and limited scalability for edge-device deployment. These limitations collectively define the core motivation of this study—developing a more efficient, interpretable, and resource-friendly model tailored for complex sensor time series classification tasks.

From a modeling perspective, shapelets and Transformer architectures address complementary aspects of sensor time series classification. Shapelets focus on capturing explicit and interpretable local discriminative patterns, whereas Transformer-based models are well suited for modeling long-range temporal dependencies and complex inter-variable interactions. This complementarity provides a natural motivation for integrating shapelet representations within a Transformer-based framework.

To address the aforementioned challenges, we propose EffiShapeFormer, a novel framework designed to substantially enhance both efficiency and classification accuracy in sensor time-series classification tasks. This framework introduces two key innovations:

(1) Dual-layer Filtering Mechanism (DFM): We propose a two-stage screening strategy, implemented as an algorithmic filtering module within the Shapelet discovery phase. In the first stage, a coarse-grained rapid screening based on Euclidean distance is performed to eliminate candidate subsequences with limited discriminative potential. In the second stage, a refined evaluation combines Perceptual Subsequence Distance (PSD) [18,19] and information gain metrics to further select highly discriminative shapelets. This dual-layer design effectively reduces computational costs while maintaining strong discriminative capability. The filtered shapelets are then modeled in a Class-specific Representation module that employs a Transformer to capture category-specific characteristics.
(2) Convolution-Inverted Attention (CIA) Module: We design a novel CIA module that integrates convolutional operations into the Transformer’s self-attention mechanism [6] to enhance local temporal pattern extraction. By inverting the attention dimension from the temporal axis to the variable axis, the module achieves bidirectional modeling of temporal and variable dependencies. This design not only reduces computational complexity but also strengthens the model’s ability to capture multi-variable interactions effectively.

The main contributions of this paper can be summarized in the following three aspects:

- We propose a Dual-layer Filtering Mechanism that significantly reduces redundant computations in the Shapelet discovery process, enhancing efficiency;
- We designed a learnable neural network module, termed Convolutional-Inverted Attention (CIA), which is integrated into the proposed model to efficiently fuse temporal and variable dependencies, thereby improving scalability and classification accuracy;
- We validated the model’s effectiveness on multiple sensor-time series datasets. Experimental results demonstrate that our approach outperforms ShapeFormer in classification accuracy, computational efficiency, and interpretability, providing a scalable and practical solution for real-world time series analysis tasks and enabling faster and more interpretable feature extraction.

The remainder of this paper is organized as follows. Section 2 reviews time series classification models and related research based on Shapelet learning; Section 3 introduces the fundamental concepts and theoretical methods of this study; Section 4 details the overall architecture and core module design of the proposed method; Section 5 presents the experimental setup and analyzes the results; Section 6 summarizes the paper and outlines the directions of our future research.

## 2. Related Work

In this section, we summarize recent advancements in time series classification tasks, highlighting the strengths and limitations of existing methods to lay the groundwork for future research.

### 2.1. Time Series Classification Model

TSC aims to identify the category to which an input sequence belongs based on its dynamic change patterns. With the advancement of deep learning, numerous neural network models have been applied to classification tasks. Early research primarily employed structures such as Convolutional Neural Networks (CNNs) [20] and Recurrent Neural Networks (RNNs) [21]. A pioneering approach like the Multi-Channel Convolutional Neural Network (MCDCNN) [22] applied CNNs to TSC. To better capture dependencies in long sequences, the Transformer architecture was introduced to time series analysis [5]. By enabling global feature interactions through its Self-Attention Mechanism, the Transformer no longer relies on strict sequence position modeling, significantly enhancing its representational power for lengthy sequences. Subsequently, numerous improved models emerged rapidly, including Informer [7], Autoformer [9], and Reformer [8] for long sequence modeling, as well as Crossformer [10] and LightTS [11] for multivariate time series. These models demonstrate superior performance in both prediction and classification tasks, further validating the potential of the Transformer architecture in time series modeling.

Beyond attention-driven models, a number of lightweight and efficient time series modeling approaches have emerged in recent years. The DLinear [23] model proposed by Zeng et al. decomposes time series into trend and seasonal components, modeling each separately through linear layers. This significantly reduces model complexity while maintaining strong predictive performance and interpretability. Wu et al.’s TimesNet [24] model transforms time series into two-dimensional images, using multi-period convolutional modules to extract pattern information across different time scales. This approach demonstrates enhanced capabilities in modeling periodicity and multi-scale structures. These methods offer novel perspectives for time series classification and, to some extent, break through the limitations of traditional deep learning models.

Despite demonstrating outstanding performance in time series classification, deep models still face significant bottlenecks due to high computational complexity and insufficient interpretability. Enhancing model transparency and efficiency has become a key research direction.

### 2.2. Shapelet-Based Time Series Methods

In recent years, research on the interpretability of time series has garnered increasing attention, with its core objective being to reveal the decision-making basis of deep models when processing temporal data [25,26,27]. Under this background, researchers have proposed various time series classification methods based on shapelets. Related studies have gradually reached a consensus: shapelets not only offer strong interpretability but also serve as a key factor in enhancing time series classification performance [13,14,15,28,29]. Early shapelet discovery methods typically enumerated all possible sub-sequences within a sequence, selecting the one with maximum information gain as a shapelet candidate [13]. This exhaustive strategy incurs extremely high computational costs. Subsequent research attempted to construct shapelets through random generation or the use of common sub-sequences [28,30]. However, such approaches often lack the correlation between positional information and variable levels, resulting in limited discriminative power [30]. Recently, the OSD [18] method and its improved variant, Shapeformer, have made significant strides in enhancing shapelet quality while reducing computational overhead. However, existing approaches still face challenges such as high computational burden and insufficient global modeling in complex sensor data scenarios. Balancing local interpretability with global dependency modeling in high-dimensional temporal data remains an urgent research challenge.

### 2.3. Other Interpretable Time Series Classification Methods

In addition to shapelet-based and attention-driven models, several other interpretable time series classification approaches have been explored in the literature. Prototype-based methods construct class-level representations using one or multiple representative time series and perform classification based on similarity matching, providing a straightforward form of interpretability [31,32]. Symbolic methods, such as Symbolic Aggregate approXimation (SAX)-based and Symbolic Fourier Approximation (SFA)-based approaches, discretize time series into symbolic representations and conduct classification in the symbolic domain, where discriminative subsequences can be explicitly identified [33,34,35]. Moreover, Convolutional Neural Network (CNN)-based models have been combined with visualization techniques, such as class activation mapping and gradient-based attribution, to highlight salient temporal regions or variables that contribute to classification decisions [36,37].

Although existing interpretable time series classification methods provide valuable insights from different perspectives, they often exhibit inherent limitations when applied to complex and high-dimensional sensor data. Prototype-based and symbolic approaches typically emphasize global similarity or rely on predefined discretization schemes, which may fail to capture fine-grained local discriminative patterns under non-stationary and noisy conditions. CNN-based interpretability methods usually provide post-hoc explanations, where interpretability is not explicitly embedded into the model structure.

In contrast, shapelets represent explicit and semantically meaningful local subsequence patterns that enable intuitive interpretation at the pattern level, while the Transformer architecture is particularly effective at modeling long-range dependencies and global interactions in multivariate time series. However, existing studies rarely integrate shapelets as explicit representations within a Transformer-based modeling framework. This observation motivates our work to incorporate shapelet representations into a Transformer architecture, aiming to jointly capture interpretable local patterns and global temporal dependencies within a unified and efficient model.

## 3. Preliminaries

### 3.1. Single-Channel/Multi-Channel Time Series Classification

We represent a time series sample as $X\in R^{L\times D}$, where *D* denotes the number of channels(variables) and *L* represents the length of the time series.All time series samples are of equal length *L*, which is obtained through standard preprocessing (e.g., sliding-window segmentation) applied to the raw sensor signals. When D=1, it represents a single-channel time series; when D>1, it represents a multi-channel time series. Here, $X=[X^{1},...,X^{D}]$, and each $X^{d}$ corresponds to a time series for channel *d*. Specifically, $X^{d}=\left[x_{1}^{d},\ldots ,x_{L}^{d}\right]$, where $x_{t}^{d}$ signifies the value of channel *d* at time step *t* within *X*. For a time series training dataset containing *N* samples, we define it as $C={\left\{(X^{\left(n\right)},y^{\left(n\right)})\right\}}_{n=1}^{N}$, where $X^{\left(n\right)}\in R^{L\times D}$ represents the *n*th time series sample, $y^{\left(n\right)}\in Y$ denotes its corresponding category label, and *Y* is the set of all labels. The Time Series Classification (TSC) task involves training a classifier $f_{\theta }:R^{L\times D}\to Y$ to predict the category of time series samples with unknown labels.

### 3.2. Shapelet

Given a time series sample $X\in R^{L\times D}$, a Shapelet $S_{i}$ is defined as a consecutive subsequence extracted from a single channel:

(1) $$S_{i}=X_{p_{i}^{start}:p_{i}^{end},d_{i}}=\left[x_{p_{i}^{start}}^{d_{i}},x_{p_{i}^{start}+1}^{d_{i}},\ldots ,x_{p_{i}^{end}}^{d_{i}}\right]\in R^{\ell_{i}},$$

where $d_{i}\in \left\{1,\ldots ,D\right\}$ is the channel index, $p_{i}^{start}$ and $p_{i}^{end}$ are the start and end indices in the source series, and $\ell_{i}$ = $p_{i}^{end}-p_{i}^{start}+1\ll L$ is the shapelet length.

In addition, we store the meta information $\left(\ell_{i},d_{i},p_{i}^{start},p_{i}^{end}\right)$ for indexing and position-related operations, while all distance computations are conducted on the numeric subsequence $S_{i}$.

### 3.3. Perceptually Important Points (PIPs)

The PIP method was first proposed in [38]. For a time series *X*, we first construct a list of PIPs and add the first and last indices of *X* to it (PIPs = [1, *L*]). Subsequently, by recursively searching the sequence for the point with the maximum perpendicular distance (Maximum Perpendicular Distance, PD) from the line formed by the first two selected PIPs, the index corresponding to this point is added as a new PIP to the list. This process is repeated until the desired number of PIPs is obtained.

### 3.4. Euclidean Distance(ED)

Given a shapelet $S_{i}\in R^{\ell_{I}}$ and an input time series *X*, we define all consecutive subsequences of length $\ell_{i}$ on the corresponding channel $d_{i}$ as:

(2) $$X_{i}^{b}=X_{b:b+\ell_{i}-1,\;d_{i}}={\left[x_{b}^{d_{i}},\;x_{b+1}^{d_{i}},\;\ldots ,\;x_{b+\ell_{i}-1}^{d_{i}}\right]}^{⊤},\;b=1,2,\ldots ,L-\ell_{i}+1.$$

The Euclidean distance between the shapelet $S_{i}$ and the time series S is defined as the minimum distance over all sliding windows:

(3) $$ED\left(S_{i},X\right)=\min_{1\leq b\leq L-\ell_{i}+1}{∥S_{i}-X_{i}^{b}∥}_{2}=\min_{1\leq b\leq L-\ell_{i}+1}\sqrt{\sum_{t=0}^{\ell_{i}-1}{\left(s_{i,t+1}-x_{b+t}^{d_{i}}\right)}^{2}},$$

where *b* denotes the sliding window start index in *X*; *t* indexes the position within a window; $x_{b+t}^{d_{i}}$ denotes the value on channel $d_{i}$ at time b+t.

### 3.5. Multi-Head Attention Mechanism (MHA) [6]

Given an input time series $X^{emb}\in R^{L\times d_{emb}}$, where $X^{emb}$ represents the input embedding matrix of the time series, linear mappings produce Query, Key, and Value matrices: $Q=XW_{Q}$, $K=XW_{K}$, $V=XW_{V}$, where $W_{Q},W_{K},W_{V}\in R^{d_{emb}\times d_{emb}}$ is a learnable projection matrix. Each head computes attention outputs in blocks across feature dimensions, defined as:

(4) $${\mathrm{head}}_{h}=\mathrm{Softmax}\left(\frac{Q_{h}K_{h}^{⊤}}{\sqrt{d_{k}}}\right)V_{h}.$$

Among these, $Q_{h},K_{h},V_{h}\in R^{L\times d_{k}}$ and ${\mathrm{head}}_{h}$ represents the output matrix of the *h*-th attention head. $\sqrt{d_{k}}$ denotes the scaling factor to stabilize gradients, and $d_{k}=d_{emb}/H$, where *H* is the number of total attention heads, Softmax(·) represents the standard softmax function of computing attention weights. The above formula can be further expanded as:

(5) $${\mathrm{head}}_{h}=\mathrm{Softmax}\left(\frac{XW_{Q}^{h}{\left(XW_{K}^{h}\right)}^{⊤}}{\sqrt{d_{k}}}\right)XW_{V}^{h}.$$

After concatenating the results from all attention heads, the final output is obtained through a linear mapping:

(6) $$\mathrm{MHA}\left(H\right)=\mathrm{concat}\left(head_{1},\ldots ,head_{H}\right)W_{O},$$

where $W_{o}\in R^{d_{emb}\times d_{emb}}$ is the output projection matrix.

In Table 1, we summarize the important notations and descriptions in the paper.

## 4. Methodology

### 4.1. Overall Architecture

**Figure 1:**
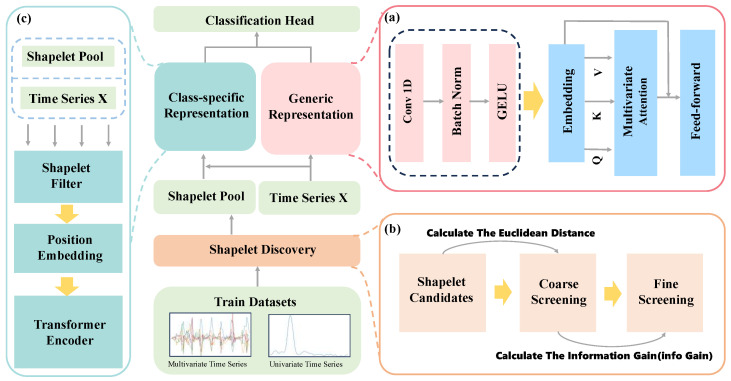
Framework of EffiShapeFormer. (**a**) Generic Representation: We propose Convolutional Inverse Attention (CIA), which employs a “dimension transposition” approach to treat variables as tokens and time points as features. This shifts the application dimension of self-attention from the temporal axis to the variable axis, significantly enhancing computational efficiency; (**b**) Shapelet Discovery: PIP is employed to extract shapelet candidates, which undergo coarse-grained screening before calculating distance and information gain to select the final shapelet; (**c**) Class-specific Representation: A self-attention-based shapelet learning mechanism captures the interactive relationship between shapelets and input sequences to learn discriminative feature representations.

Figure 1 presents the overall methodological framework proposed in this study. To address the high computational overhead introduced by information gain calculations during Shapelet candidate selection in the original Shapelet discovery module, as well as the low training efficiency of the Transformer’s self-attention mechanism when processing large-scale time series data, we introduce two key structural enhancements to the ShapeFormer model. These improvements are designed to significantly boost both computational efficiency and temporal modeling performance.

During the Shapelet discovery phase, we design a coarse screening strategy based on Euclidean distance to rapidly eliminate candidate subsequences with weak discriminative capability prior to detailed evaluation. This strategy significantly reduces the frequency of distance computations and information gain evaluations, thereby substantially decreasing the time cost of Shapelet mining.

In the general representation learning module, we propose a novel Convolution-Inverted Attention (CIA) neural network module. This design replaces the original two-layer convolutional structure with a single-layer convolutional architecture, thereby enhancing computational efficiency while retaining strong local feature extraction capability. Moreover, by introducing an inverse attention mechanism that shifts the computation dimension of self-attention from the temporal axis to the variable axis, the model can effectively capture inter-variable dependencies. This approach substantially reduces training time while preserving the model’s discriminative performance. The following sections will detail the specific modules of our method.

### 4.2. Coarse Screening in Shapelet Discovery

**Figure 2:**
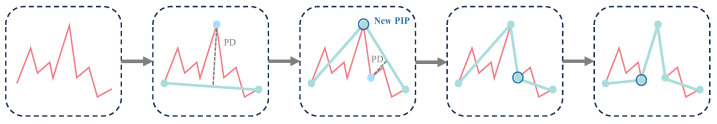
The process of extracting first 5 PIPs. In that, PD is maximum Perpendicular Distance.

In the Shapelet Discovery module, we improved the Offline Shapelet Discovery (OSD) method. During the shapelet candidates extraction phase, we employed Perceptually Important Points (PIPs) to extract Shapelets from the training set $C={\left\{\left(X^{\left(n\right)},y^{\left(n\right)}\right)\right\}}_{n=1}^{N}$ [38]. Specifically, we recursively search the time series *X* for the newest PIP with the maximum vertical distance from the line formed by two previously selected PIPs. When a new PIP is added to the PIP set, we use the third consecutive PIP to obtain new shapelet candidates. Thus, for a new PIP, up to three Shapelets may be added to the shapelet candidates set [19,39]. In this paper, we adopt the same strategy as Shapeformer [19], setting the number of PIPs to $n_{pip}=0.2\times L$ and *L* as the time series length, selecting up to $3\times n_{pip}$ shapelet candidates. Each shapelet simultaneously stores its numerical segment, start and end positions, and associated variable channel information, providing data support for subsequent segment screening. Figure 2 shows an example of identifying the first 5 PIPs from the time series *X* in the training dataset.

Although the PIP method effectively reduces the number of shapelet candidates, the computational burden remains significant during subsequent screening due to the need for repeated PSD and information gain calculations. To address this issue, this paper proposes a coarse-grained screening mechanism based on Euclidean distance. This approach is grounded in two key considerations: First, Euclidean distance itself is computationally straightforward, making it suitable for rapid preliminary screening of large-scale shapelet candidates. Second, from the perspective of shape similarity, Euclidean distance effectively reflects the discriminative potential of shapelet candidates. Although Euclidean distance is known to be sensitive to noise and scaling shifts, its use in the coarse-grained screening phase is justified by the fact that this stage focuses on rapidly filtering out obviously non-discriminative shapelets from large datasets. Since this phase is preliminary, the impact of noise is minimized as it only serves to reduce the pool of shapelet candidates. Additionally, by using more refined methods, such as information gain, in the subsequent fine-grained screening phase, we ensure that only the most discriminative shapelets are selected. Therefore, the use of Euclidean distance in the coarse screening phase effectively enhances the overall efficiency of the shapelet discovery process without significantly compromising the classification accuracy.

**Figure 3:**
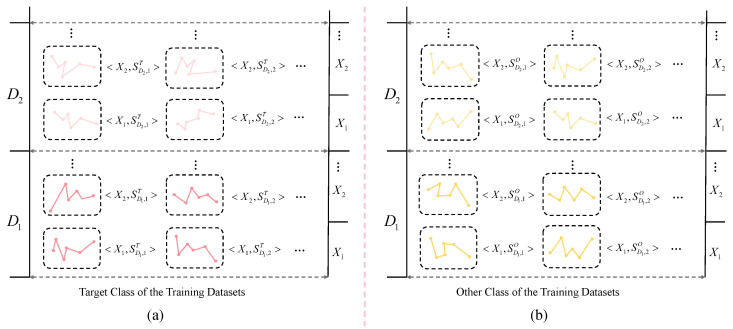
Representative examples of shapelet candidates in the training datasets. (**a**) illustrates the shapelets extracted from the target class, while (**b**) presents those extracted from the other classes.

By employing the coarse screening mechanism to eliminate less discriminative candidates before fine-grained screening, we significantly enhance the overall efficiency of the discovery process. For ease of presentation in the coarse-grained screening stage, we introduce $S_{D_{j},k}^{T}$ and $S_{D_{j},k}^{O}$ to denote shapelet candidates indexed by channel $D_{j}$ and candidate index *k* in the target and other classes, respectively. This is only an indexing notation and does not change the shapelet definition in Section 3; each $S_{D_{j},k}^{\left(\cdot \right)}$ still corresponds to a numeric shapelet subsequence extracted from a single channel, together with its meta information (length and location). Consequently, all distance computations in this section are performed on the same numeric subsequences; the superscripts/subscripts are used solely for bookkeeping and for describing the coarse screening process succinctly. We categorize shapelet candidates extracted from the training set $C={\left\{\left(X^{\left(n\right)},y^{\left(n\right)}\right)\right\}}_{n=1}^{N}$ into two classes: $<X_{i},S_{D_{j},k}^{T}>$ represents shapelet candidates on $X_{i}$ within the target class, while $<X_{i},S_{D_{j},k}^{O}>$ denotes shapelet candidates on $X_{i}$ within the other class, as illustrated in Figure 3. For $<X_{i},S_{D_{j},k}^{T}>,i=1,...,n_{C}^{T},k=1,...,3n_{pip}$, $n_{C}^{T}$ indicates the number of samples in the target class, *m* denotes the variable index, and $3n_{pip}$ represents the Shapelet index. For $<X_{i},S_{D_{j},k}^{O}>,i=1,...,n_{C}^{O},k=1,...,3n_{pip}$, $n_{C}^{O}$ denotes the number of samples in the other class. Accordingly, $<X_{i},S_{D_{j},k}^{\left(\cdot \right)}>$ simply denotes evaluating candidate $S_{D_{j},k}^{\left(\cdot \right)}$ on sample $X_{i}$ during screening.

**Figure 4:**
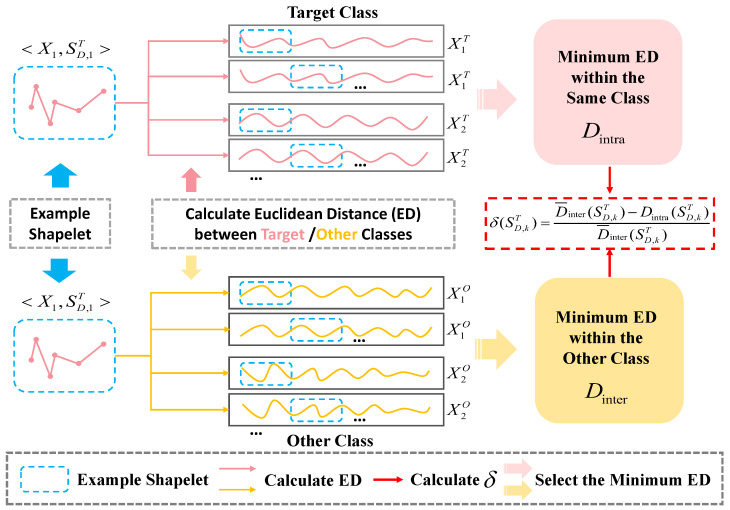
Calculate the intra-class distance and inter-class distance between shapelets and the training data *X* during the coarse screening process.

Our coarse screening process is illustrated in Figure 4. For a given target class candidate shapelet $S_{D,k}^{T}$ and time series sample $X_{i}$, their minimum Euclidean distances within the target class and across other classes are defined as follows:

(7) $$D_{\mathrm{intra}}\left(S_{D,k}^{T}\right)=\underset{i=1,...,n_{C}^{T}}{min}\mathrm{ED}\left(S_{D,k}^{T},X_{i}\right),$$

(8) $$D_{\mathrm{inter}}\left(S_{D,k}^{T}\right)=\underset{i=1,...,n_{C}^{O}}{min}\mathrm{ED}\left(S_{D,k}^{T},X_{i}\right),$$

where $n_{C}^{T}$ and $n_{C}^{O}$ are the numbers of samples from the target class and from other classes, respectively; and ED(·,·) is the minimum Euclidean distance (Equation (3)).

Calculate the average minimum distance ${\bar{D}}_{\mathrm{inter}}\left(S_{D,k}^{T}\right)$ of this shaplet across other categories, then define a discriminative metric $\delta (S_{D,k}^{T})$ based on the distance differences between samples of different categories for filtering.

(9) $$\delta \left(S_{D,k}^{T}\right)=\frac{{\bar{D}}_{\mathrm{inter}}\left(S_{D,k}^{T}\right)-D_{\mathrm{intra}}\left(S_{D,k}^{T}\right)}{{\bar{D}}_{\mathrm{inter}}\left(S_{D,k}^{T}\right)}.$$

A larger δ(·) indicates that the candidate is more discriminative for separating the target class from other classes. We rank candidates by δ(·) in descending order and discard the bottom β% candidates, where β is an experimental hyperparameter, for which we conducted hyperparameter sensitivity experiments in Section 5.3.

After the coarse screening process concludes, the retained shapelet candidates are designated as $S=\{S_{1},...,S_{G}\}$ and enter the Fine Screening module. By calculating their Perceptual Subsequence Distance (PSD) with all instances in the training data $X\in R^{L\times D}$, the optimal information gain is identified to evaluate their discriminative capability. The Shapelet set $S^{\prime }$ with the highest information gain is selected as the final choice and stored in the Shapelet pool.

(10) $$\mathrm{PSD}\left(X,S_{i}\right)=\min_{b=1,\ldots ,\;L-\ell_{i}+1}\mathrm{CID}\left(X_{b:b+\ell_{i}-1,\;d_{i}},\;S_{i}\right),$$

here, *b* denotes the sliding window start index in *X* (not the start index of the shapelet in its source seties), $d_{i}$ and $\ell_{i}$ are the channel index and length of $S_{i}$, $X_{b:b+\ell_{i}-1,\;d_{i}}$ is the length -$\ell_{i}$ subsequence on channel $d_{i}$ starting at *b*; and CID(·,·) signifies the complexity-invariant distance.

By introducing a correction factor related to the intrinsic pattern complexity of the sequence, this metric effectively enhances the robustness of traditional Euclidean distance in measuring morphological similarity. It has been demonstrated to improve the discriminative capability of shapelets in time series classification tasks [40].

### 4.3. Class-Specific Representation

To deeply mine discriminative features highly correlated with categories within time series, we introduced a class-specific representation module into our model. Based on the self-attention mechanism of Transformers, this module constructs high-level feature representations by modeling the differential relationships between shapelets and input sequences.

Each $S_{i}^{\prime }$ in the final shapelet set $S^{\prime }$ records its length $\ell_{i}^{\prime }$, channel index $d_{i}$, and position $\left(P_{i}^{start},P_{i}^{end}\right)$ within the original sequence. For input sequence *X*, we compute $S_{i}^{\prime }$ distances between all subsequences in *X* on channel $d_{i}$, restricting the search range to a neighborhood centered at $P_{i}^{start}$ with radius *w*. The subsequence with the shortest distance becomes the best-fit subsequence $I_{i}$.

(11) $$J_{i}=\underset{b\in W(p_{i}^{start},w)}{argmin}\mathrm{CID}\left(X_{b:b+\ell_{i}^{\prime }-1,d_{i}},S_{i}^{\prime }\right),$$

(12) $$I_{i}=X_{J_{i}:J_{i}+\ell_{i}^{\prime }-1,d_{i}}.$$

We linearly project both the shapelet $S_{i}^{\prime }$ and its best-fit subsequence $I_{i}$ into the same embedding space $h_{s_{i}^{\prime }}=P_{S}\left(S_{i}^{\prime }\right)$, $h_{I_{i}}=P_{I}\left(I_{i}\right)$, yielding their difference features: $F_{i}=h_{I_{i}}-h_{s_{i}^{\prime }}$. Here, $F_{i}\in R^{d_{speci}}$ and P(·) denotes the linear projection, while $d_{speci}$ represents the embedding size of the difference features. Subsequently, the difference features $F_{i}$ are integrated with position embeddings to capture their sequential order. To better indicate the positional information of shapelets, both the position index $p_{i}^{start},P_{i}^{end}$ and channel index $d_{i}$ of shapelets are learned through linear projection to obtain their embeddings.

(13) $${\tilde{F}}_{i}=F_{i}+\mathrm{PE}\left(p_{i}^{start}\right)+\mathrm{PE}\left(p_{i}^{end}\right)+\mathrm{PE}\left(d_{i}\right).$$

Here, PE(·) is the position embedding function, which maps the start point, end point, and one-hot encoded variables into dense vectors via a learnable linear projection, thereby endowing the model with positional awareness.

Feed all ${\tilde{F}}_{i}\in R^{1\times d_{speci}}$ into the MHA of the Transformer Encoder, where *G* denotes the number of elements in $S^{\prime }$. Given the projection $W_{Q},W_{K},W_{V}\in R^{h\times d_{speci}\times \left(d_{speci}/H\right)}$, compute the attention weight for position *i* to position *j*, ultimately yielding the output $Z^{speci}=\left\{Z_{1}^{speci},\ldots ,Z_{G}^{speci}\right\}$, where $Z_{i}^{speci}\in R^{d_{speci}}$.

(14) $$\alpha_{i,j}=\mathrm{Softmax}\left(\frac{\left({\tilde{F}}_{i}W_{Q}\right){\left({\tilde{F}}_{j}W_{K}\right)}^{⊤}}{\sqrt{d_{speci}}}\right),$$

(15) $$Z_{i}^{speci}=\sum_{j=1}^{G}\alpha_{i,j}\left({\tilde{F}}_{i}W_{V}\right).$$

Due to the category-specific nature of these features, attention scores between samples of the same category are significantly higher than those between samples of different categories, thereby enhancing the model’s ability to distinguish between categories. Simultaneously, leveraging the local discriminative properties of shapelet, differential features can identify representative key subsequences across different time segments and variable dimensions within the time series. This enables the model to more effectively capture temporal dependencies and cross-variable correlations within the sequence.

### 4.4. Generic Representation

To enhance the effectiveness of modeling multivariate time series features, we propose a novel universal feature extraction module—CIA (Convolution-Inverted Attention)—whose overall structure is illustrated in Figure 1a. The core concept of the CIA module is to achieve synergistic integration between local feature extraction and global variable correlation modeling. Traditional Transformers compute attention over the temporal dimension, which can capture long-term dependencies but incurs high computational overhead and tends to overlook inherent correlations between variables. Conversely, while convolutional operations efficiently extract local temporal patterns, their limited receptive field makes it difficult to model global dependencies.

**Figure 5:**
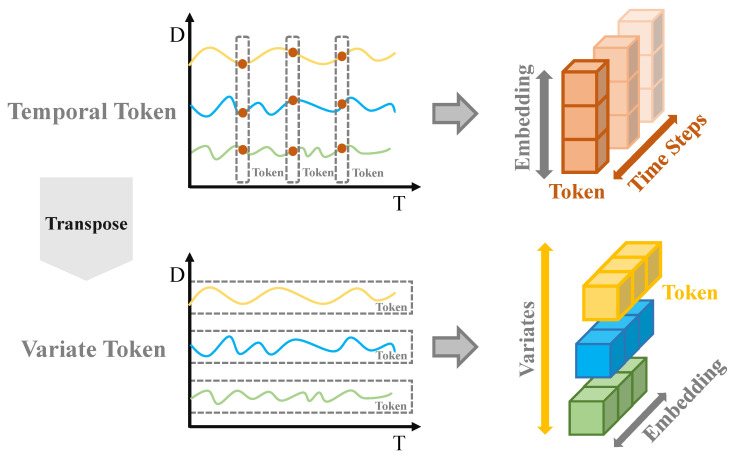
Token Dimension Conversion.

Inspired by iTransformer [41], this module employs a dimensional Conversion approach, treating variables as tokens and time points as features. This shifts the application dimension of Self-Attention from the temporal axis to the variable axis, as illustrated in Figure 5. This design enables the model to explicitly learn correlations between variables while leveraging one-dimensional convolutional layers to efficiently capture local morphological features in the temporal dimension. The CIA module achieves dual modeling of temporal and variable dependencies while maintaining computational efficiency, significantly enhancing the discriminative power and generalization capabilities of the general representation.

Unlike traditional iTransformer, the CIA module incorporates convolutional layers into the self-attention mechanism. The convolution operation allows the CIA module to achieve a stronger local receptive field, improving its ability to capture local temporal patterns. Additionally, the convolutional layers help reduce the computational cost, making the model more efficient when handling long time series. In contrast, iTransformer only inverts the attention dimension to model dependencies between time and variables, without incorporating convolution, limiting its ability to efficiently extract local features.This design is particularly important for sensor time series, which often exhibit strong local fluctuations, short-term transient patterns, and noise-contaminated dynamics. By introducing a convolutional layer before inverted attention, the CIA module explicitly captures local temporal variations that are typically under-modeled by the purely attention-based iTransformer, while preserving its ability to model global inter-variable dependencies.

One-Dimensional Convolution for Local Feature Extraction: For the time series $X\in R^{L\times D}$, we employ a convolutional module for local feature extraction. This convolutional block consists of a one-dimensional convolutional layer (Conv1D), batch normalization (BatchNorm), and a GELU activation function in sequence. The computational process is as follows:

(16) U=GELU(BatchNorm(Conv1D(X))).

The kernel dimensions of the convolution are $R^{1\times d_{c}}$, where $d_{c}$ is the kernel size of the convolution filter. The resulting universal features are $U\in R^{L\times d_{gener}}$, where $d_{gener}$ is the feature dimension of the convolved output, which controls the subsequent number of tokens.

**Figure 6:**
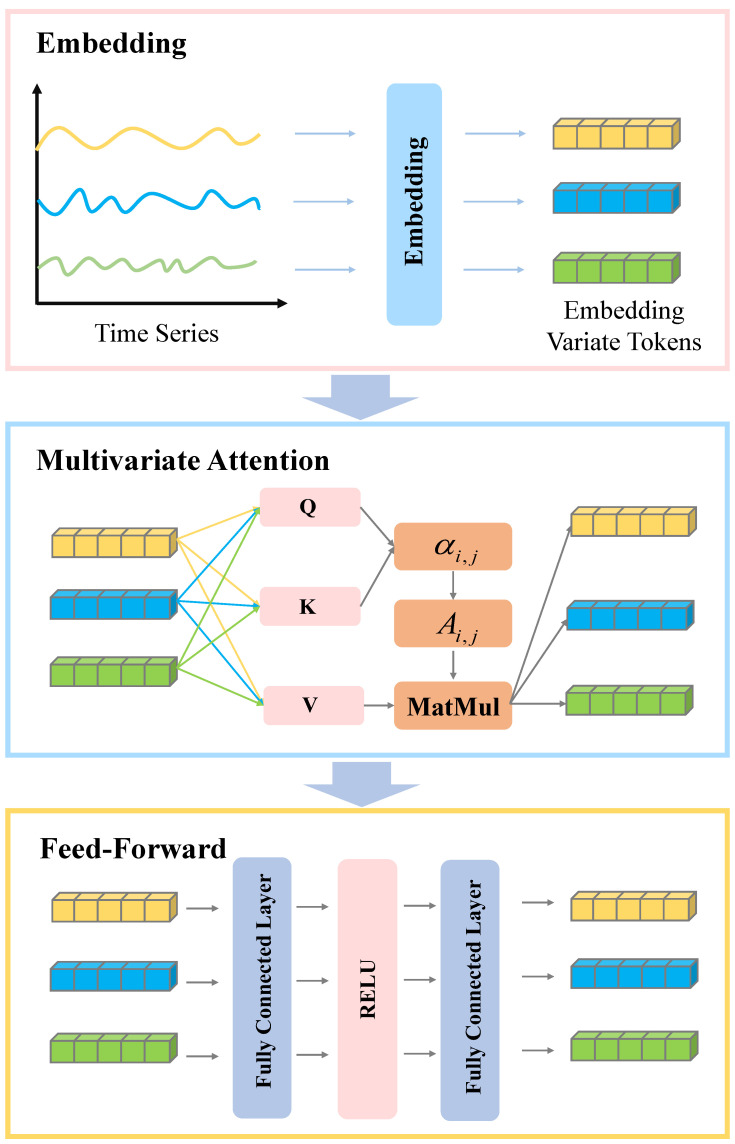
Overall Structure of Inverse Attention.

Inverse Attention Models Variable Dependencies: The overall structure is shown in Figure 6. After obtaining features containing local information *U*, we transpose dimensions to treat variables as tokens and time points as features: $E=U^{⊤}+P\in R^{d_{gener}\times L}$, where $P\in R^{d_{gener}\times L}$ is the learnable position encoding. To convert time series embeddings into variable token representations, we employ a Multi-layer Perception (MLP) network to map each variable’s time series embedding to dimension $d_{var}$. This transforms each variable into a token [41,42], $E^{\prime }=\mathrm{MLP}\left(E\right)\in R^{d_{gener}\times d_{var}}$, where $d_{var}$ represents the mapping dimension. Consequently, we obtain $d_{gener}$ variable tokens.Subsequently, feature $E^{\prime }$ is input into the multi-head attention mechanism to learn correlations. Through the linear projection matrix $W_{Q},W_{K},W_{V}\in R^{d_{var}\times d_{var}}$, queries, keys, and values $(Q=E^{\prime }W_{Q},K=E^{\prime }W_{K}$, $V=E^{\prime }W_{V}\in R^{d_{gener}\times d_{var}}$) are obtained. $q_{i},k_{i}\in R^{d_{var}}$ serves as the query and key for a variable token. For any pair of variable tokens i,j, their pre-Softmax score is:

(17) $$A_{i,j}=\frac{q_{i}^{⊤}k_{j}}{\sqrt{d_{var}}}.$$

The correlation between variable *i* and variable *j* in the projection is measured by $\alpha_{i,j}$, expressed in matrix form as:

(18) $$A=\frac{QK^{⊤}}{\sqrt{d_{var}}}\in R^{d_{gener}\times d_{gener}}.$$

Next, the Softmax function yields the weight coefficients $\alpha_{i,:}=\mathrm{Softmax}\left(A_{i,:}\right)\in R^{d_{gener}}$. These weights are then applied to sum all values, resulting in the output $E^{gener}=\left[E_{1}^{gener},\ldots ,E_{d_{gener}}^{gener}\right]\in R^{d_{gener}\times d_{var}}$,

(19) $$E_{i}^{gener}=\sum_{j=1}^{d_{gener}}\alpha_{i,j}V_{j}\in R^{d_{var}}.$$

After obtaining the variable representation $H^{gener}$ updated through the self-attention mechanism, the model further performs independent nonlinear mapping on the features of each variable token via a Feed-Forward Network (FFN) [6] to enhance its expressive capability. This process employs residual connections and Layer Normalization to maintain training stability.

(20) $$\tilde{E}=\mathrm{LayerNorm}\left(E^{gener}\right),$$

(21) $$Z^{gener}=\mathrm{LayerNorm}\left(\tilde{E}+\mathrm{FFN}\left(\tilde{E}\right)\right)\in R^{d_{gener}\times d_{var}},$$

among these, the FFN(·) consists of two fully connected layers and the GELU activation function, which performs a nonlinear feature transformation on each variable token. Since this module uses classical features as input tokens, we employ average pooling to derive the final class tokens:

(22) $$Z^{gener}=\mathrm{AvgPooling}\left(Z^{gener}\right).$$

Under this architecture, the self-attention weight matrix clearly reflects global correlations among variables, thereby enhancing model interpretability. The final output $Z^{gener}\in R^{d_{var}}$ effectively integrates local temporal patterns with global variable dependencies.

### 4.5. Classification Head

To synergistically leverage feature information across different levels, this model concatenates category-specific representations $Z^{speci}$ with general representations $Z^{gener}$ to form a fused representation $Z^{con}$ as input to the classification head. This fusion strategy enables the model to make more robust classification decisions by simultaneously utilizing global variable correlations from the general representations and local discriminative patterns revealed by Shapelet within the category-specific representations.

(23) $$Z^{con}=\mathrm{concat}\left(Z^{speci},Z^{gener}\right).$$

### 4.6. Big-O Complexity Analysis

In this section, we provide an analysis of the computational complexity of the proposed modules (DFM, CIA, Transformer encoder) to support the claims of improved efficiency. The complexity of each module is evaluated using Big-O notation, allowing for a clear understanding of the performance improvements over previous methods. Table 2 illustrates the complexity analysis for each module in EffiShapeFormer.

The **DFM** module involves two stages: Coarse Screening and Fine Screening. The Coarse Screening stage uses Euclidean distance calculations, which have a complexity of O(L·N), where *L* is the time series length and *N* is the number of shapelet candidates. The Fine Screening stage incorporates Perceptual Subsequence Distance (PSD), which involves $O(L^{2})$ operations due to the pairwise distance computations.

The **CIA** module modifies the Transformer self-attention mechanism by shifting the attention dimension from the temporal axis to the variable axis. The quadratic complexity of the self-attention mechanism is $O(D^{2}\cdot L)$, where *D* is the number of variable and *L* is the length of the sequence.

The **Transformer Encoder**’s self-attention mechanism has a complexity of $O(L^{2}\cdot D)$, where *L* is the sequence length and *D* is the dimensionality of the input.

By integrating the DFM and the CIA module, our model achieves a significant reduction in computational complexity, especially in comparison to previous methods such as ShapeFormer. The overall complexity of the EffiShapeFormer framework is reduced from $O(L^{2}\cdot D)$ to $O(L\cdot N+D^{2}\cdot L)$, since D≪L, this demonstrates the efficiency improvements we have achieved.

## 5. Experience

### 5.1. Experimental Settings

#### 5.1.1. Datasets

This study evaluates the proposed method using three types of sensor time series datasets. One category employs single-channel time series datasets from the UCR [43] Archive. The UCR Archive comprises 85 distinct time series classification datasets covering various types such as bio-signals, action recognition, speech signals, and sensor signals. It stands as one of the most widely used benchmark libraries in time series classification research. We selected thirteen sensor-related datasets for experimentation, with most implementations mirroring configurations from prior studies.

The second category utilizes the UEA Archive multi-channel time series datasets. Comprising over 31 multi-channel time series classification datasets, the UEA [44] Archive spans diverse application scenarios including mechanical fault detection, human action recognition, medical signal analysis, and sensor monitoring. It stands as one of the most commonly used benchmark libraries in multi-channel time series classification research. We selected five datasets related to mechanical sensors for experimentation, with most implementations mirroring configurations employed in other studies.

The third category employs the Gearbox Dataset [45], a multi-channel mechanical dataset from Southeast University. This dataset was acquired from the Drivetrain Dynamic Simulator (DDS) and comprises two sub-datasets: bearings and gears. Data for four fault types was collected for bearings and gears under two operating conditions (speed-load configurations of 20-0 and 30-2). Fault type descriptions are shown in the table. Each file contains 8 signals representing: 1— motor vibration; 2, 3, 4—Vibration of the planetary gearbox in the x, y, and z directions; 5—Motor torque; 6, 7, 8—Vibration of the parallel gearbox in the x, y, and z directions. Table 3 is the descriptions for Bearingset and Gearset.

#### 5.1.2. Data Preprocessing

The UCR [43] and UEA [44] datasets has been split into training and testing portions, with most components ready for direct experimentation. A validation set was selected from each training dataset using an 80/20 ratio. However, the DodgerLoopDay dataset contained a small number of missing values (NaN), which we repaired using mean imputation.

In this experiment, we primarily preprocessed the Gearbox [45] Dataset. Due to the lengthy time series for each fault type in both the bearing and gear sub-datasets, we reduced the computational burden while maintaining data representativeness by truncating each sequence to sixty-fourth of its original length. We then performed non-overlapping sampling using a sliding window size of 1024. Subsequently, the first 80% of each fault category was used as the training set, and the remaining 20% as the test set. The validation set was processed identically to the UCR and UEA datasets. The processed bearing and gear data were then categorized into two operating conditions, yielding the final four datasets.

Beyond truncation and sliding-window sampling, no additional preprocessing (e.g., filtering, artifact removal, or normalization) was applied to preserve original signal characteristics and maintain format consistency with the UCR and UEA datasets. Detailed information for each data set is presented in Table 4.

#### 5.1.3. Implementation Details

Our model was trained using the RAdam optimizer with an initial learning rate of $5\times 10^{-2}$ and weight decay of $5\times 10^{-4}$. The training process involved 64 batch sizes and ran for 200 epochs, with all other parameters consistent with Shapeformer. To ensure experimental fairness, we set the window size and number of extracted shapelets to 100 and 10, respectively, (except for the SonyAIBORobotSurface1, SonyAIBORobotSurface2, Libras, ERing and RacketSports datasets, where the window size was set to 10) for both Shapeformer and our method. Before experiments, we performed hyperparameter tuning. After final hyperparameters were determined, model training and testing proceeded. Training employed early stopping based on validation set loss. All experiments were implemented in PyTorch 2.2.2 on Python 3.10.18. (Computational Infrastructure: Windows operating system, GPU NVIDIA GeForce RTX 4090 with 24 GB VRAM (NVIDIA Corporation, Santa Clara, CA, USA)).

#### 5.1.4. Baselines

To validate the effectiveness and advanced nature of the proposed method, we selected the most representative time series models currently available as comparative benchmarks. Baseline methods for time series classification are summarized as follows:

(1) **Autoformer** [9]: A time series Transformer based on autocorrelation mechanisms, capturing long-term dependencies through trend-seasonal decomposition to enhance long-sequence prediction performance.
(2) **Crossformer [10]**: Models dependencies among multivariate time series via cross-dimensional attention mechanisms, enabling efficient learning of full-dimensional interactive features.
(3) **DLinear [23]**: A linear decomposition-based time series modeling method achieving efficient forecasting through independent modeling of trend and seasonal components.
(4) **Informer [7]**: An efficient Transformer employing ProbSparse self-attention and hierarchical distillation architecture for long-sequence time series forecasting.
(5) **iTransformer [41]**: Replaces traditional time-dimension modeling with feature-dimension modeling for more efficient multivariate time series representation learning.
(6) **LightTS [11]**: A lightweight time series model constructed using a simple MLP architecture combined with two downsampling strategies: interval sampling and continuous sampling. This approach leverages the observation that “time series downsampling often preserves key information,” significantly reducing computational complexity while maintaining accuracy.
(7) **PatchTST [46]**: Inputs time series divided into local patches into a Transformer, enhancing local pattern capture and prediction stability.
(8) **Reformer [8]**: An efficient Transformer variant that introduces locality-sensitive hashing (LSH) attention and reversible layers to significantly reduce memory and computational complexity, enabling scalable modeling of long time series sequences.
(9) **Shapeformer [19]**: Combines shapelet feature extraction with the Transformer architecture to learn shape-aware representations for time series.
(10) **TimesNet [24]**: Maps one-dimensional time series to two-dimensional tensors, modeling periodicity and local variations through multi-scale convolutions in a two-dimensional time-frequency space for universal temporal feature extraction.

In all baseline experiments, we strictly adhere to the parameter configurations specified in their original papers. Validation loss-based early stopping is employed throughout training to ensure fairness and comparability of experimental results.

#### 5.1.5. Evaluation Metrics

To comprehensively evaluate the performance of the proposed method, this study employs multiple classification evaluation metrics, including Accuracy (ACC) and F1-Score (F1). We computed the average values of these metrics for each model and conducted a comprehensive ranking based on these averages to measure the overall classification performance. Additionally, to validate the computational efficiency of the model, we recorded the shapelet discovery time and total training time for both our method and the Shapeformer model under the same task for efficiency comparison.

(24) $$\mathrm{ACC}=\left(\frac{\mathrm{TP}+\mathrm{TN}}{\mathrm{TP}+\mathrm{FP}+\mathrm{FN}+\mathrm{TN}}\right),$$

(25) $$F1=\left(\frac{2\times \mathrm{PR}\times \mathrm{RE}}{\mathrm{PR}+\mathrm{RE}}\right).$$

In these equations, T, F, P, and N represent true, false, positive, and negative, respectively. For example, TP denotes the number of true positives, while FN denotes the number of false negatives.

### 5.2. Experimental Results

#### 5.2.1. Performance Evaluation

To comprehensively evaluate the performance of the proposed EffiShapeFormer method, we conducted systematic comparisons with baseline approaches across all experimental datasets. A total of ten representative baseline models were selected, and their classification accuracy (Accuracy) and F1-Score were recorded. The main experimental results are presented in Table 5, Table 6, Table 7 and Table 8. For ease of comparison, the best and second-best results are highlighted in bold and underlined, respectively.

As shown in Table 5 and Table 6, the proposed EffiShapeFormer model achieves significant performance improvements over the baseline ShapeFormer in terms of average classification accuracy, yielding an approximate 6% increase. This improvement highlights the superior feature extraction and representation capabilities of our proposed framework. Furthermore, EffiShapeFormer consistently ranks first among all baseline methods, indicating its strong adaptability and robustness across diverse datasets.

In terms of the F1-Score metric, as presented in Table 7 and Table 8, EffiShapeFormer also demonstrates superior performance, surpassing ShapeFormer by approximately 5.6% and achieving the highest overall average F1-Score among all comparative models. These results verify that EffiShapeFormer not only improves the classification precision but also maintains a better balance between precision and recall, reflecting its enhanced ability to handle imbalanced and complex time series patterns.

Although our method does not attain the best performance on every single dataset, it achieves Top-1 accuracy on 12 datasets and Top-2 accuracy on 2 datasets, demonstrating excellent generalization ability, stability, and competitiveness across multiple evaluation scenarios. Overall, these experimental results strongly validate the effectiveness and robustness of the proposed model in sensor-based time series classification tasks.

It can be observed from Table 5, Table 6, Table 7 and Table 8 that no single method consistently achieves the best performance across all datasets. This variability is closely related to the diverse characteristics of the evaluated datasets, as summarized in Table 4.

EffiShapeFormer demonstrates particularly strong performance on datasets with longer sequence lengths, multiple sensor channels, and clear local discriminative patterns, such as Bearing20/30, Gear20/30, Epilepsy, and SonyAIBORobotSurface. In these scenarios, the proposed Dual-layer Filtering Mechanism effectively selects informative shapelets, while the CIA module jointly captures local temporal dynamics and cross-variable dependencies, which aligns well with the intrinsic structure of multivariate sensor signals.

In contrast, for datasets with very short sequences, extremely limited training samples, or relatively weak local temporal structure, simpler models or methods with stronger inductive biases toward global similarity may occasionally achieve slightly better results. This observation is consistent with prior studies and highlights that dataset characteristics such as dimensionality, sequence length, and class complexity play a critical role in determining model effectiveness.

Overall, although EffiShapeFormer does not dominate every individual dataset, it achieves the best average performance across all evaluated benchmarks, indicating its robustness and adaptability across diverse sensor time series classification scenarios.

#### 5.2.2. Hyperparameter Stability

In this method, an important hyperparameter—the coarse screening threshold β—needs to be adjusted. To analyze the impact of this parameter on model performance and verify its stability, we conducted systematic experiments across all datasets using different values of β to evaluate the model’s classification performance. Specifically, the coarse screening threshold was set within the range [0.05, 0.10, 0.15, 0.20, 0.25, 0.3, 0.4, 0.5, 0.6, 0.7] to investigate the model’s sensitivity to this parameter and identify a suitable value that balances efficiency and performance. Table 9 summarizes the optimal threshold obtained for each sensor dataset. Under these threshold settings, the model achieved the highest accuracy and F1-Score on the corresponding dataset. Therefore, in subsequent experiments, we adopted these optimal threshold values for model training and evaluation. It is worth noting that β is selected offline for each dataset based on training/validation performance. Once determined, the corresponding β is fixed and consistently used throughout training and testing on that dataset, without any further adjustment at inference time.

Specifically, the choice of β depends on the characteristics of the dataset: (1) In datasets with higher noise levels or stronger non-stationarity, the discriminative distance distribution of candidate shapelets is more dispersed. In such cases, a larger β is required to avoid prematurely discarding potentially useful shapelets. (2) In datasets with strong intra-class consistency and clear inter-class differences, the coarse screening phase can reliably distinguish candidate shapelets, and a smaller β is sufficient to reduce computational cost while maintaining performance. (3) Variations in sequence length and channel dimensions across different datasets can also affect the number and statistical properties of shapelet candidates, thereby influencing the optimal choice of β.

Therefore, the variability of β across datasets is not due to instability in the method, but rather reflects the differences in the density of shapelet discriminative information under different data distributions.

#### 5.2.3. Computational Efficiency Analysis

In the proposed method, a Coarse Screening module is incorporated during the shapelet discovery stage, and a Convolutional-Inverted Attention (CIA) module is integrated within the generic representation stage. Compared with ShapeFormer model, our approach achieves a higher overall computational efficiency while maintaining strong classification performance. To verify this advantage, we conducted a systematic comparative analysis of both shapelet discovery time and model training time on eight datasets.

**Figure 7:**
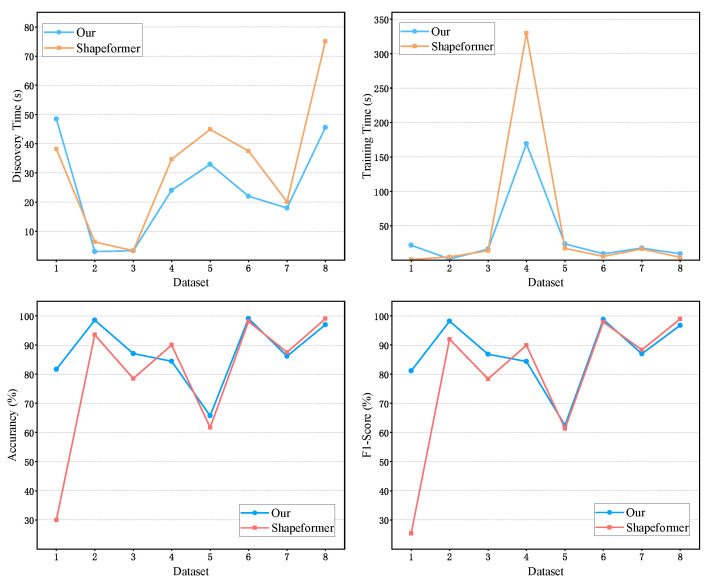
Comparison of discovery time, training time, accuracy, and F1-Score between our method and Shapeformer on eight datasets.The horizontal axis denotes the dataset indices: 1—Car, 2—DodgerLoopWeekend, 3—ERing, 4—Libras, 5—Lightning7, 6—Plane, 7—RacketSports, 8—Trace.

**Shapelet Discovery Time.** The shapelet discovery time refers to the total duration spent during the shapelet discovery stage, encompassing both the extraction and filtering processes. As shown in Figure 7, our method consistently requires less discovery time than ShapeFormer on eight datasets. This improvement can be attributed to the introduced Coarse Screening mechanism, which effectively eliminates redundant or non-discriminative shapelet candidates in the early stage, thereby reducing unnecessary computation. The experimental results clearly demonstrate that the Coarse Screening module plays a crucial role in enhancing shapelet discovery efficiency and significantly accelerates the overall shapelet discovery process.

**Model Training Time.** To further evaluate the effectiveness of the CIA module in reducing computational costs during model training, we compared the training times of our method and ShapeFormer on eight datasets. As illustrated in Figure 7, our method exhibits substantially lower training time than ShapeFormer. This result indicates that the CIA module efficiently captures cross-class feature representations while reducing redundant parameter updates, thereby significantly lowering the computational burden during training. Overall, the results confirm that the proposed framework achieves an excellent balance between computational efficiency and classification performance.

### 5.3. Ablation Study

To further verify the effectiveness and contribution of each module in the proposed method, we conducted a systematic ablation study on all datasets. Specifically, we progressively removed or added key modules while keeping other structures unchanged, and recorded the model’s average Accuracy and F1-Score under different configurations. This approach enables a quantitative assessment of each module’s impact on the overall performance improvement. All comparisons were made with respect to ShapeFormer baseline, thereby revealing the specific role of each component in enhancing the model’s discriminative capability and feature representation.

As shown in Table 10, the baseline ShapeFormer achieves an Accuracy of 0.7855 and an F1-Score of 0.7634 without any additional components. When the Coarse Screening module is introduced, the performance slightly decreases to an Accuracy of 0.7675 and an F1-Score of 0.7510, suggesting that coarse screening mainly reduces the interference of irrelevant shapelets but, when used alone, provides limited direct gains for classification. When incorporating the inverse-attention mechanism into ShapeFormer, the performance improves to an Accuracy of 0.7937 and an F1-Score of 0.7832, indicating that inverse attention can better model informative dependencies and enhance feature discrimination. Building upon this, the Convolutional-Inverted Attention (CIA) module further boosts the performance to an Accuracy of 0.8113 and an F1-Score of 0.7849, demonstrating that combining convolutional feature projection with inverse-attention-based interaction is more effective than using inverse attention alone. Finally, the Proposed Model, which integrates both the Coarse Screening mechanism and the CIA module, achieves the best overall performance with an Accuracy of 0.8456 and an F1-Score of 0.8263, confirming the complementary contributions of the proposed components while maintaining computational efficiency.

### 5.4. A Case Study of Epilepsy

**Figure 8:**
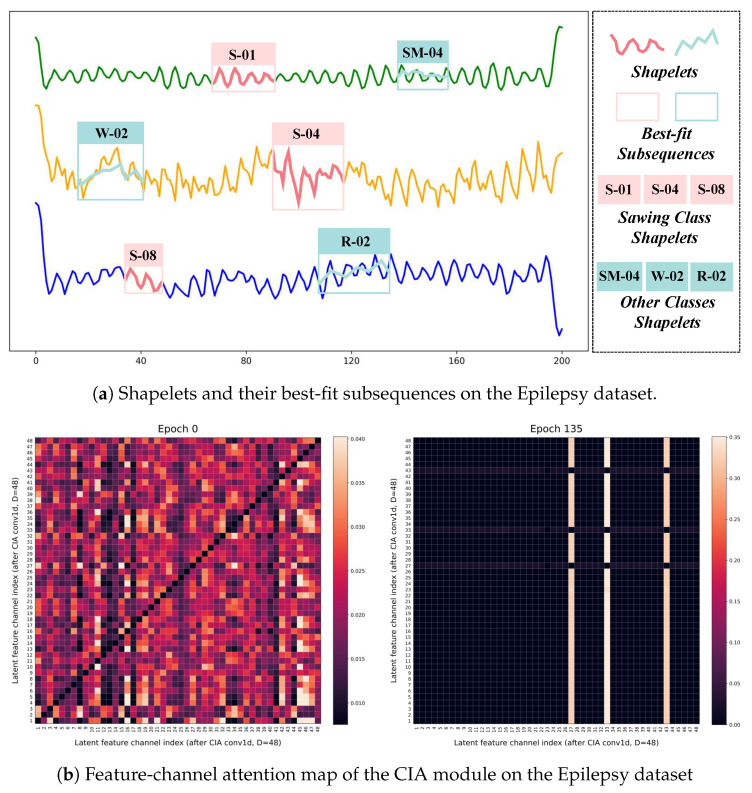
(**a**) Shapelets and their best-fit subsequences on the Epilepsy dataset (a randomly selected Sawing instance). Pink: top three Sawing shapelets (S-01, S-04, S-08); cyan: top shapelets from other classes (SM-04, W-02, R-02). Shaded boxes indicate the corresponding best-fit subsequences. (**b**) Feature-channel attention map of the CIA module on the Epilepsy dataset (Epoch 0 vs. Epoch 135). Diagonal entries are masked (shown as zero) for visualization only to emphasize cross-channel attention.

To interpret the results of EffiShapeFormer, we adopt the Epilepsy dataset from the UEA archive [44], which contains four activity classes (Running, Walking, Sawing, and Seizure Mimicking) for human activity recognition. Each class consists of three channels. For each class, we select 10 shapelets for analysis. Specifically, we randomly choose a Sawing instance from the training set and select the top three shapelets from this class. Meanwhile, we select one top shapelet from each of the other three classes for comparative visualization. The results are shown in Figure 8a. In this figure, S, R, W, and SM denote the Sawing, Running, Walking, and Seizure Mimicking classes, respectively. The suffix-01 (or -04) indicates the shapelet index within the corresponding class. For instance, S-01 refers to the first shapelet in the Sawing class, and SM-04 refers to the fourth shapelet in the Seizure Mimicking class. The outlined boxes indicate the corresponding best-fit subsequences matched by each shapelet. As can be observed, EffiShapeFormer is able to localize key subsequences across different channels and temporal positions and match them with the learned shapelets. Compared with shapelets from other classes, the same class shapelets exhibit higher similarity to their best-fit subsequences, highlighting the model’s ability to capture class-discriminative local patterns in time series.

As shown in Figure 8b, we visualize the channel-wise attention response of the CIA module in EffiShapeFormer on the Epilepsy dataset, and compare the attention distributions at the early training stage (Epoch 0) and after convergence (Epoch 135). The rows and columns of the attention matrix correspond to the 48 latent feature channels produced by the CIA conv1d projection (the convolution mapping dimension is set to 48 in our experiments). On top of these latent channels, an inverse-attention mechanism is applied to model cross-channel dependencies. Specifically, each element $A_{i,j}$ denotes the attention weight assigned to channel *j* when updating the representation of channel *i*, thereby characterizing the strength of inter-channel interactions.

To emphasize cross-channel relations and avoid self-correlation dominating the visualization, the diagonal entries are masked and shown as zero for plotting purposes only. At Epoch 0, the attention pattern is relatively diffuse and lacks stable structure, indicating that the model has not yet formed consistent cross-channel dependencies. In contrast, at Epoch 135, the attention map exhibits clear stripe-like structures: several bright vertical stripes suggest that a small subset of channels is consistently attended by many other channels, behaving as key channels in cross-channel interactions; meanwhile, some horizontal bands indicate that certain channels rely consistently on specific key channels during their updates. Overall, the converged attention evolves from an unstructured diffuse pattern to a sparser and more organized one, suggesting that conv1d provides compact latent channel representations, while inverse attention further promotes effective cross-channel dependencies and suppresses redundant interactions. This supports the capability of EffiShapeFormer to capture discriminative dependency patterns in the latent feature space.

## 6. Conclusions

In this study, we propose an efficient model architecture named EffiShapeFormer for sensor-based time series classification tasks. The model introduces a dual filtering mechanism in the Shapelet Discovery stage to efficiently select discriminative shapelets. In the Class-specific Representation module, the filtered shapelets are modeled to capture class-specific features, while in the Generic Representation stage, the proposed CIA module is employed to extract cross-class generic features. Finally, the model fuses the class-specific and generic representations to achieve efficient and accurate time series classification, significantly improving computational efficiency while maintaining high accuracy.

The ablation experiments verify the functional contributions of the proposed modules: the Coarse Screening module effectively reduces computational time during the shapelet discovery phase while maintaining high accuracy; the CIA module accelerates the training process and better captures global dependencies among variables. The combination of both modules not only enhances the overall efficiency of the model but also further improves classification performance.

Experimental results on 22 sensor datasets demonstrate that EffiShapeFormer achieves the best overall average performance and consistently competitive results compared with baseline models, highlighting the effectiveness and potential of our approach for sensor-based time series classification tasks. In future work, we plan to further exploit the interpretability of shapelets to explore their broader applicability across various sensor time series analysis scenarios.

## Figures and Tables

**Table 1:** Important Notations and Descriptions.

Notation	Description
*N*	The number of training samples
$x_{t}^{d}$	Value at time step *t* on channel *d*
$S_{i}$	The *i*-th shapelet (a continuous subsequence from one channel)
$d_{i}$	The channel index of shapelet $S_{i}$
$p_{i}^{\mathrm{start}},\;p_{i}^{\mathrm{end}}$	Start/end indices of shapelet $S_{i}$
$\ell_{i}$	The length of shapelet $S_{i}$
*b*	The sliding-window start index in matching
$X_{i}^{b}=X_{b:b+\ell_{i}-1,\;d_{i}}$	Length-$\ell_{i}$ subsequence on channel $d_{i}$ starting at *b*
ED(·,·)	Euclidean distance
$n_{pip}$	The number of PIPs
$S_{D_{j},k}^{\left(T\right)},S_{D_{j},k}^{\left(O\right)}$	The candidate indexed by channel $D_{j}$ and candidate id *k*, from target class (*T*) or other class (*O*). (Does not redefine shapelets.)
$n_{C}^{T},n_{C}^{O}$	The number of samples in target class/other classes during coarse screening
$D_{\mathrm{intra}}\left(\cdot \right),D_{\mathrm{inter}}\left(\cdot \right)$	The minimum intra/inter-class distance of shapelet
δ(·)	Discriminative score based on intra/inter-class distance difference for coarse screening
β	Percentage threshold: discard bottom β% candidates ranked by δ(·)
S	Candidate set retained after coarse screening
$S_{i}^{\prime }$	The *i*-th final shapelet after fine screening
PSD(·,·)	Perceptual subsequence distance based on CID(·,·)
CID(·,·)	Complexity-invariant distance
$I_{i}$	Best-fit subsequence in *X* aligned to $S_{i}^{\prime }$
$h_{S_{i}^{\prime }},h_{I_{i}}$	Embeddings of shapelet $S_{i}^{\prime }$ and matched subsequence $I_{i}$ via linear projections
PE(·)	Learnable embedding function for $p_{i}^{start},p_{i}^{end},d_{i}$
*w*	Neighborhood radius for local matching around $p_{i}^{\mathrm{start}}$
$Z_{\mathrm{speci}},\;Z_{\mathrm{gener}},\;Z_{\mathrm{con}}$	Class-specific, generic, and concatenated representations
H,h	Number of attention heads; index of the *h*-th head
Softmax(·)	Softmax function for attention normalization

**Table 2:** Complexity Analysis for Each Module in EffiShapeFormer.

Module	Complexity	Explanation
DFM	O(L·N)	Coarse Screening involves Euclidean distance calculation; Fine Screening uses Perceptual Subsequence Distance (PSD).
CIA	$O(D^{2}\cdot L)$	The modified self-attention mechanism reduces complexity by shifting the attention dimension.
Transformer Encoder	$O(L^{2}\cdot D)$	Traditional Transformer self-attention mechanism with quadratic complexity.
EffiShapeFormer	$O(L\cdot N+D^{2}\cdot L)$	Combined efficiency of DFM and CIA, reducing the overall computational cost.

**Table 3:** Fault type descriptions for Bearingset and Gearset.

Location	Type	Description
Gearset	Chipped	Crack occurs in the gear feet
	Miss	Missing one of feet in the gear
	Root	Crack occurs in the root of gear feet
	Surface	Wear occurs in the surface of gear
Bearingset	Ball	Crack occurs in the ball
	Inner	Crack occurs in the inner ring
	Outer	Crack occurs in the outer ring
	Comb	Crack occurs in the both inner and outer ring

**Table 4:** Characteristics of the Datasets. # denotes number of.

Datasets	#Channels	Series Length	Num Classes	#Train	#Val	#Test
Bearing20	8	1024	4	163	41	48
Bearing30	8	1024	4	163	41	48
Car	1	577	4	48	12	60
DodgerLoopDay	1	288	7	62	16	80
DodgerLoopGame	1	288	2	16	4	138
DodgerLoopWeekend	1	1500	2	240	60	600
Earthquakes	1	512	2	257	65	139
Epilepsy	3	207	4	110	27	138
ERing	4	65	6	24	6	270
Handwriting	3	152	26	120	30	850
Libras	2	45	15	144	36	180
Gear20	8	1024	4	163	41	48
Gear30	8	1024	4	163	41	48
Lightning2	1	637	2	48	12	61
Lightning7	1	319	7	56	14	73
Plane	1	144	7	84	21	105
RacketSports	6	30	4	121	30	152
SonyAIBORobotSurface1	1	70	2	16	4	601
SonyAIBORobotSurface2	1	65	2	21	6	953
StarLightCurves	1	1024	3	800	200	8236
Trace	1	275	4	80	20	100
Wafer	1	152	2	800	200	6164

**Table 5:** Comparison of Classification Accuracy on Datasets (Part 1).

Dataset	Autoformer	Crossformer	DLinear	Informer	iTransformer	LightTS
Bearing20	0.2917	0.4583	0.2500	0.5000	0.3542	0.3125
Bearing30	0.2083	0.5625	0.6042	0.5208	0.5417	0.5833
Car	0.2833	0.6833	0.7833	0.7333	0.8000	0.8000
DodgerLoopDay	0.2987	0.6234	0.5584	**0.6494**	0.5065	0.6104
DodgerLoopGame	0.5197	0.4409	0.6535	0.4803	0.5118	0.6929
DodgerLoopWeekend	0.6349	0.0714	0.9524	0.9683	0.9762	0.9683
Earthquakes	0.5540	0.6835	0.6835	0.7410	**0.7554**	0.7194
Epilepsy	0.7681	0.8623	0.4565	0.7899	0.6739	0.8406
ERing	0.6370	0.9444	0.8963	0.9481	0.9296	0.9000
Handwriting	0.0529	0.1859	0.1365	0.2024	0.2035	0.1306
Libras	0.7167	0.8556	0.6722	0.6611	0.8611	0.6722
Gear20	0.2708	0.7917	0.5833	0.9792	0.4583	0.5833
Gear30	0.2292	0.7500	0.7708	0.8333	0.7500	0.6875
Lightning2	0.5082	0.7377	0.6721	0.7377	0.7213	0.6885
Lightning7	0.2192	0.6712	0.6712	0.7397	0.6164	0.6712
Plane	0.9524	0.9619	0.9810	0.9524	0.9714	0.9714
RacketSports	0.7697	0.7632	0.7500	**0.8882**	0.7434	0.6711
SonyAIBORobotSurface1	0.4409	0.6606	0.5957	0.4293	0.4759	0.4210
SonyAIBORobotSurface2	0.8594	0.8562	0.8562	0.8153	0.8363	0.8468
StarLightCurves	0.2736	0.8932	0.8985	0.8981	0.8565	0.9157
Trace	0.5900	0.7700	0.5200	0.8800	0.5200	0.5500
Wafer	0.9849	0.9933	0.9429	0.9927	0.9935	0.9940
Average-ACC	0.5029	0.6918	0.6768	0.7428	0.6844	0.6923
Rank	11	7	9	4	8	6

**Table 6:** Comparison of Classification Accuracy on Datasets (Part 2).

Dataset	PatchTST	Reformer	Shapeformer	TimesNet	Our
Bearing20	0.0833	0.5833	0.7500	0.6458	**0.8744**
Bearing30	0.1250	0.5625	0.9791	0.5208	**0.9841**
Car	0.7833	0.7000	0.3000	0.7667	**0.8167**
DodgerLoopDay	0.4675	0.5325	0.4875	0.5195	0.6000
DodgerLoopGame	0.5197	0.6535	0.6304	0.4252	**0.8623**
DodgerLoopWeekend	0.7619	0.9841	0.9348	0.9841	**0.9855**
Earthquakes	0.7122	0.6547	0.7050	0.6043	0.7482
Epilepsy	0.9638	0.8333	0.9565	0.8478	**0.9783**
ERing	**0.9593**	0.9296	0.7852	0.9185	0.8704
Handwriting	0.1318	0.2388	**0.2671**	0.2106	0.2365
Libras	0.7444	0.6889	**0.9000**	0.7667	0.8444
Gear20	0.0833	**1.0000**	0.7500	0.8333	0.8333
Gear30	0.4167	0.8542	**1.0000**	0.7500	**1.0000**
Lightning2	0.6885	0.6721	**0.7869**	0.7213	**0.7869**
Lightning7	0.7123	**0.7671**	0.6164	0.6575	0.6575
Plane	0.9714	0.9619	0.9810	0.9619	**0.9905**
RacketSports	0.7237	0.8553	0.8750	0.8289	0.8618
SonyAIBORobotSurface1	0.5607	0.4326	0.8869	0.4293	**0.9018**
SonyAIBORobotSurface2	0.8468	**0.8751**	0.7964	0.8059	0.8741
StarLightCurves	0.9230	0.9023	0.9105	0.8685	**0.9299**
Trace	0.9400	0.8300	**0.9900**	0.8800	0.9700
Wafer	0.9950	0.9919	0.9924	0.9943	**0.9964**
Average-ACC	0.6415	0.7502	0.7855	0.7246	**0.8456**
Rank	10	3	2	5	1

**Table 7:** Comparison of Classification F1-Score on Datasets (Part 1).

Dataset	Autoformer	Crossformer	DLinear	Informer	iTransformer	LightTS
Bearing20	0.2561	0.4833	0.2500	0.3750	0.3182	0.3155
Bearing30	0.1735	0.4932	0.5684	0.4161	0.4958	0.5417
Car	0.2516	0.6896	0.7876	0.7455	0.8058	0.8032
DodgerLoopDay	0.2733	0.5949	0.5494	**0.6467**	0.5139	0.5872
DodgerLoopGame	0.4826	0.4319	0.6486	0.4517	0.3385	0.6851
DodgerLoopWeekend	0.5279	0.0667	0.9426	0.9611	0.9706	0.9611
Earthquakes	0.4712	0.5080	**0.6602**	0.4744	0.4818	0.5007
Epilepsy	0.7646	0.8579	0.3925	0.7724	0.6549	0.8341
ERing	0.6189	0.9440	0.8940	0.9472	0.9294	0.8984
Handwriting	0.0388	0.1569	0.0939	0.1664	0.1652	0.0887
Libras	0.6886	0.8515	0.6576	0.6500	0.8549	0.6608
Gear20	0.2594	0.7840	0.5611	0.9791	0.4405	0.5611
Gear30	0.1509	0.7412	0.7353	0.8298	0.7487	0.6792
Lightning2	0.4089	0.7208	0.6611	0.7289	0.6796	0.6799
Lightning7	0.1769	0.6115	0.6152	0.7345	0.5801	0.6151
Plane	0.9437	0.9614	0.9804	0.9518	0.9703	0.9703
RacketSports	0.7757	0.7768	0.7614	**0.8939**	0.7531	0.6816
SonyAIBORobotSurface1	0.3228	0.6519	0.5688	0.3003	0.3859	0.2986
SonyAIBORobotSurface2	0.8460	0.8510	0.8465	0.8096	0.8266	0.8322
StarLightCurves	0.2621	0.8326	0.8612	0.8698	0.6294	0.8768
Trace	0.5484	0.7421	0.4504	0.8753	0.5059	0.4846
Wafer	0.9609	0.9827	0.8317	0.9808	0.9830	0.9844
Average-F1	0.4638	0.6697	0.6508	0.7073	0.6378	0.6609
Rank	11	6	8	4	9	7

**Table 8:** Comparison of Classification F1-Score on Datasets (Part 2).

Dataset	PatchTST	Reformer	Shapeformer	TimesNet	Our
Bearing20	0.1067	0.5145	0.7433	0.6016	**0.8695**
Bearing30	0.1483	0.4932	0.9791	0.5025	**0.9837**
Car	0.7857	0.7071	0.2544	0.7632	**0.8109**
DodgerLoopDay	0.4678	0.5364	0.4830	0.4966	0.5992
DodgerLoopGame	0.3931	0.6472	0.6157	0.4200	**0.8597**
DodgerLoopWeekend	0.5542	0.9802	0.9202	0.9802	**0.9815**
Earthquakes	0.4600	0.5723	0.5476	0.5822	0.4280
Epilepsy	0.9642	0.8133	0.9567	0.8290	**0.9779**
ERing	**0.9594**	0.9283	0.7832	0.9176	0.8686
Handwriting	0.0929	0.1946	**0.2490**	0.1753	0.2128
Libras	0.7425	0.6805	**0.8992**	0.7592	0.8436
Gear20	0.0645	**1.0000**	0.7500	0.8222	0.8179
Gear30	0.3354	0.8525	**1.0000**	0.6500	**1.0000**
Lightning2	0.6655	0.6611	0.7711	0.7058	**0.7860**
Lightning7	0.6735	**0.7742**	0.6133	0.6476	0.6245
Plane	0.9703	0.9614	0.9783	0.9622	**0.9876**
RacketSports	0.7344	0.8625	0.8830	0.8370	0.8699
SonyAIBORobotSurface1	0.5200	0.3068	0.8797	0.3003	**0.9008**
SonyAIBORobotSurface2	0.8324	0.8668	0.7861	0.7936	**0.8681**
StarLightCurves	0.8948	0.8611	0.8850	0.8007	**0.9314**
Trace	0.9359	0.8235	**0.9890**	0.8753	0.9671
Wafer	0.9869	0.9784	0.9802	0.9853	**0.9908**
Average-F1	0.6040	0.7280	0.7703	0.7003	**0.8263**
Rank	10	3	2	5	1

**Table 9:** Coarse Screening Threshold β for Experimental Dataset.

Dataset	β	Dataset	β	Dataset	β
Bearing20	0.20	ERing	0.10	RacketSports	0.60
Bearing30	0.50	Handwriting	0.60	SonyAIBORobotSurface1	0.10
Car	0.40	Libras	0.05	SonyAIBORobotSurface2	0.25
DodgerLoopDay	0.25	Gear20	0.50	StarLightCurves	0.40
DodgerLoopGame	0.50	Gear30	0.30	Trace	0.10
DodgerLoopWeekend	0.50	Lightning2	0.25	Wafer	0.20
Earthquakes	0.70	Lightning7	0.10		
Epilepsy	0.60	Plane	0.40		

**Table 10:** Comparative Performance Evaluation of Proposed Model and Ablation Variants on All Experimental Datasets. The best results are highlighted in bold.

Components	Accuracy	F1-Score
ShapeFormer (Baseline)	0.7855	0.7634
Coarse Screening + ShapeFormer	0.7675	0.7510
Inverse Attention + ShapeFormer	0.7937	0.7832
CIA + ShapeFormer	0.8113	0.7849
Proposed Model	**0.8456**	**0.8263**

## Data Availability

The sensor data used in this study are publicly available from the UEA, UCR, and Gearbox databases.
